# Hypertrophic obstructive cardiomyopathy complicated with acute myocardial infarction and diffuse fibrosis: surgery or not?

**DOI:** 10.1186/s12872-022-02602-z

**Published:** 2022-04-13

**Authors:** Yunhong Wang, Xuemei Zhao, Mei Zhai, Yan Huang, Qiong Zhou, Yuhui Zhang, Yi Mao, Jian Zhang

**Affiliations:** Heart Failure Center, State Key Laboratory of Cardiovascular Disease, Fuwai Hospital, National Center for Cardiovascular Diseases, Chinese Academy of Medical Sciences and Peking Union Medical College, 167 North Lishi Road, Beijing, 100037 People’s Republic of China

**Keywords:** Myocardial infarction in the absence of obstructive coronary artery disease, Hypertrophic obstructive cardiomyopathy, Extreme hypertrophy, Late gadolinium enhancement

## Abstract

**Background:**

Hypertrophic cardiomyopathy with extreme hypertrophy, biventricular obstruction and diffuse myocardial fibrosis complicated by myocardial infarction in the absence of obstructive coronary artery disease (MINOCA) is a rare phenotype. Evidence and guideline recommendations are still lacking for a treatment strategy.

**Case presentation:**

Emergency coronary angiography was performed in a 38-year-old man with a 2-year history of nonobstructive hypertrophic cardiomyopathy (HCM) presenting with acute myocardial infarction. The coronary angiogram yielded no stenotic lesions but showed a diffusely dilated left descending artery with slow blood flow. All evidence from biomarker analysis, electrocardiography, echocardiography, and imaging supported the diagnosis of acute myocardial infarction in the left ventricular anterior wall. The echocardiogram demonstrated severe interventricular and apical hypertrophy, severe left ventricular outflow tract obstruction and mild right ventricular outflow tract obstruction. Cardiac magnetic resonance imaging showed a concentric morphological subtype of HCM with diffuse late gadolinium enhancement in the left ventricle. Extended septal myectomy was performed 1 month later, and the patient recovered well.

**Conclusions:**

Hypertrophic obstructive cardiomyopathy with acute myocardial infarction is an indication for coronary angiography. Septal reduction surgery could be performed cautiously in HCM patients with extreme hypertrophy, biventricular obstruction and diffuse myocardial fibrosis complicated by MINOCA to improve the patient’s symptoms.

## Background

Myocardial infarction in the absence of obstructive coronary artery disease is termed MINOCA and accounts for 5% to 6% of all acute myocardial infarction (AMI) patients who are referred for coronary angiography [1, 2]. MINOCA is a dynamic and descriptive diagnosis [3, 4]. The proposed underlying causes of MINOCA include plaque disruption, coronary embolus/thrombus, spontaneous coronary artery dissection, coronary artery spasm, and microvascular disease [3]. The underlying causes should be explored to guide patient treatment.

MINOCA in patients with hypertrophic cardiomyopathy (HCM) is rare [5–7]. The underlying mechanisms include small-vessel disease with decreased vasodilator capacity, septal perforator artery compression, myocardial bridging, obstruction to left ventricular outflow, inadequate capillary density in relation to the increased myocardial mass, and coronary artery spasm [8–12]. Left ventricular outflow tract (LVOT) obstruction is independently associated with enhanced thrombin generation and platelet activity in patients with HCM, likely accounting for microembolism in the coronary artery [13]. In summary, the causes are multifactorial and complicated and depend on the individualized phenotype, making it more challenging to choose the treatment strategy.

We report a case of hypertrophic obstructive cardiomyopathy (HOCM) with extreme hypertrophy, biventricular obstruction, and diffuse myocardial fibrosis that presented as MINOCA and was treated by extended septal myectomy. Thus, we provide a successful treatment paradigm for patients with HOCM complicated with acute myocardial infarction and diffuse fibrosis.

## Case presentation

A 38-year-old man who did not smoke or use illicit drugs and who had a 2-year history of HCM was admitted to the emergency room at our hospital because of chest discomfort. Two hours before admission, he felt a 5-min period of chest tightness when he squatted down to fix a faucet. One hour later, at rest, he experienced a second episode of chest tightness and pressure, which radiated to the shoulders, complicated by nausea and sweating. The discomfort was moderate to severe in intensity. This episode lasted for approximately 2 h. He denied having any palpitations or loss of consciousness during this episode.

In the past year, he had several episodes of amaurosis when he changed positions. He did not take any home medications after the diagnosis of HCM. His father died of HCM in his 50 s. His older brother was diagnosed with nonobstructive HCM by echocardiography.

**Fig. 1:**
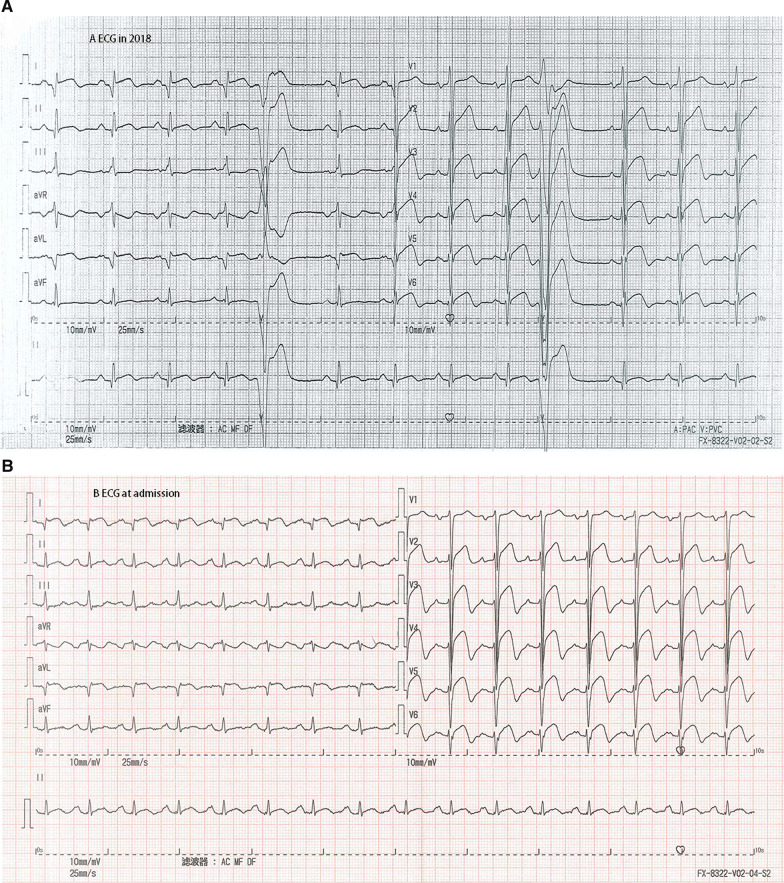

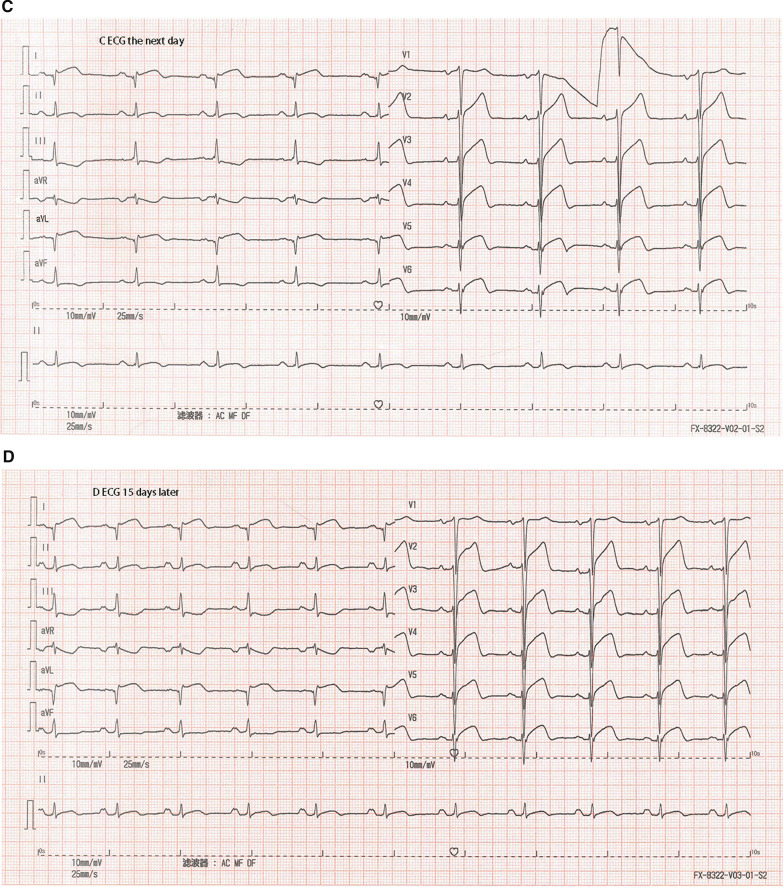
Electrocardiogram (ECG). The ECG showed persistent ST-segment elevation in precordial V1-V6 leads in 2018 and 15 days after AMI (**a**, **d**). ST-T segment changes were observed in the acute phase, including at admission and the next day (**b**, **c**). Premature ventricular contractions were observed in the first ECG in 2018 (**a**)

**Fig. 2:**
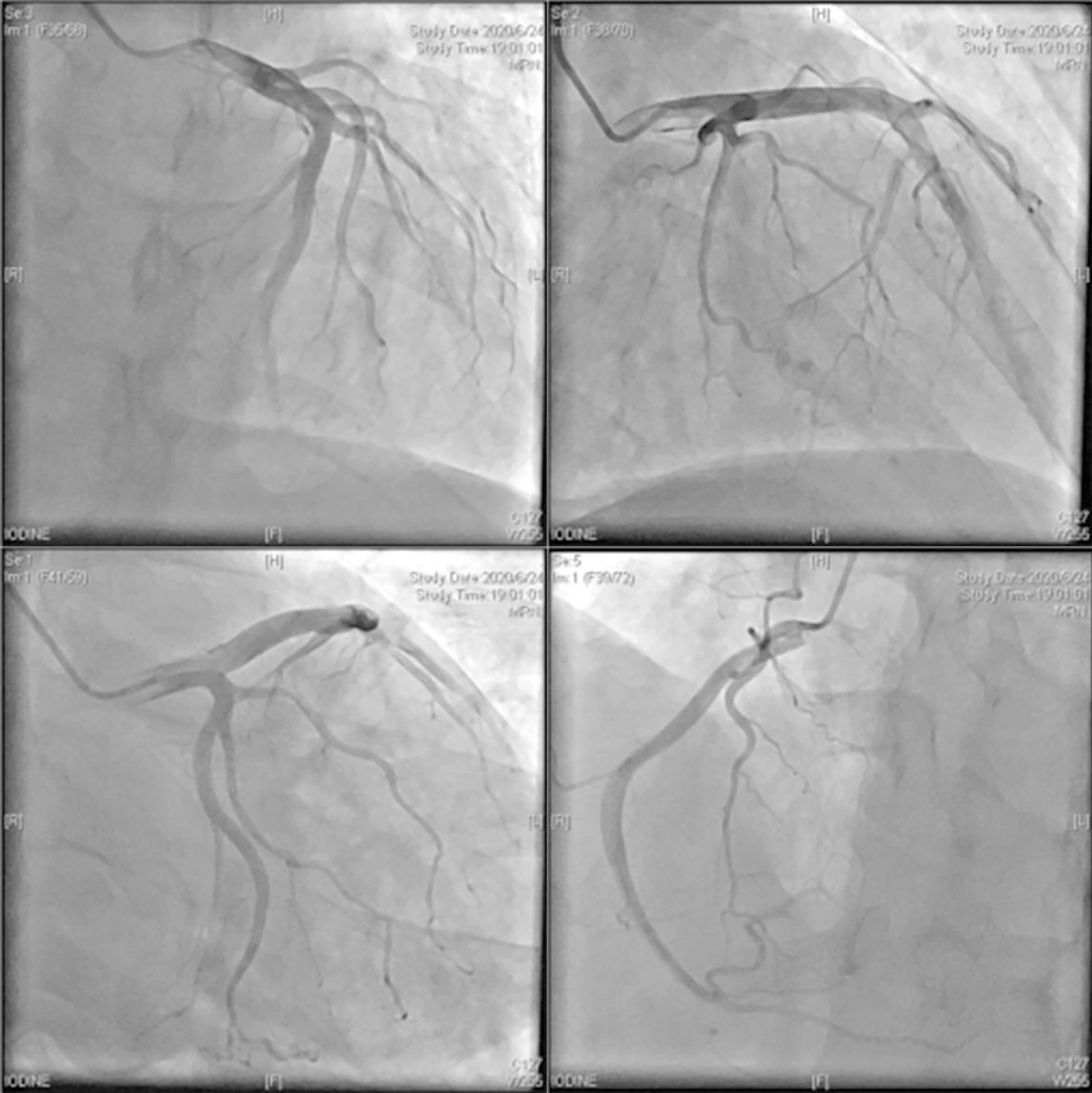
Coronary angiogram. Coronary angiography demonstrated diffuse dilation and slow blood flow of the left descending artery with no stenotic lesions

The patient’s blood pressure was 92/75 mmHg (right arm, supine position), and his heart rate was 100 bpm. The initial cardiac troponin I level was 0.126 ng/ml. The electrocardiogram (ECG) showed a normal sinus rhythm with precordial ST elevation, as well as poor R wave progression and T wave inversions in the precordial and I/aVL leads. ST-T segment changes were noted in the acute phase compared with his prior electrocardiogram in 2018 (Fig. 1). Emergency coronary angiography was performed. However, no occluding lesions in the coronary arteries were found. Instead, diffuse dilation and slow blood flow (TIMI grade II) of the left descending artery were observed (Fig. 2).

**Fig. 3:**
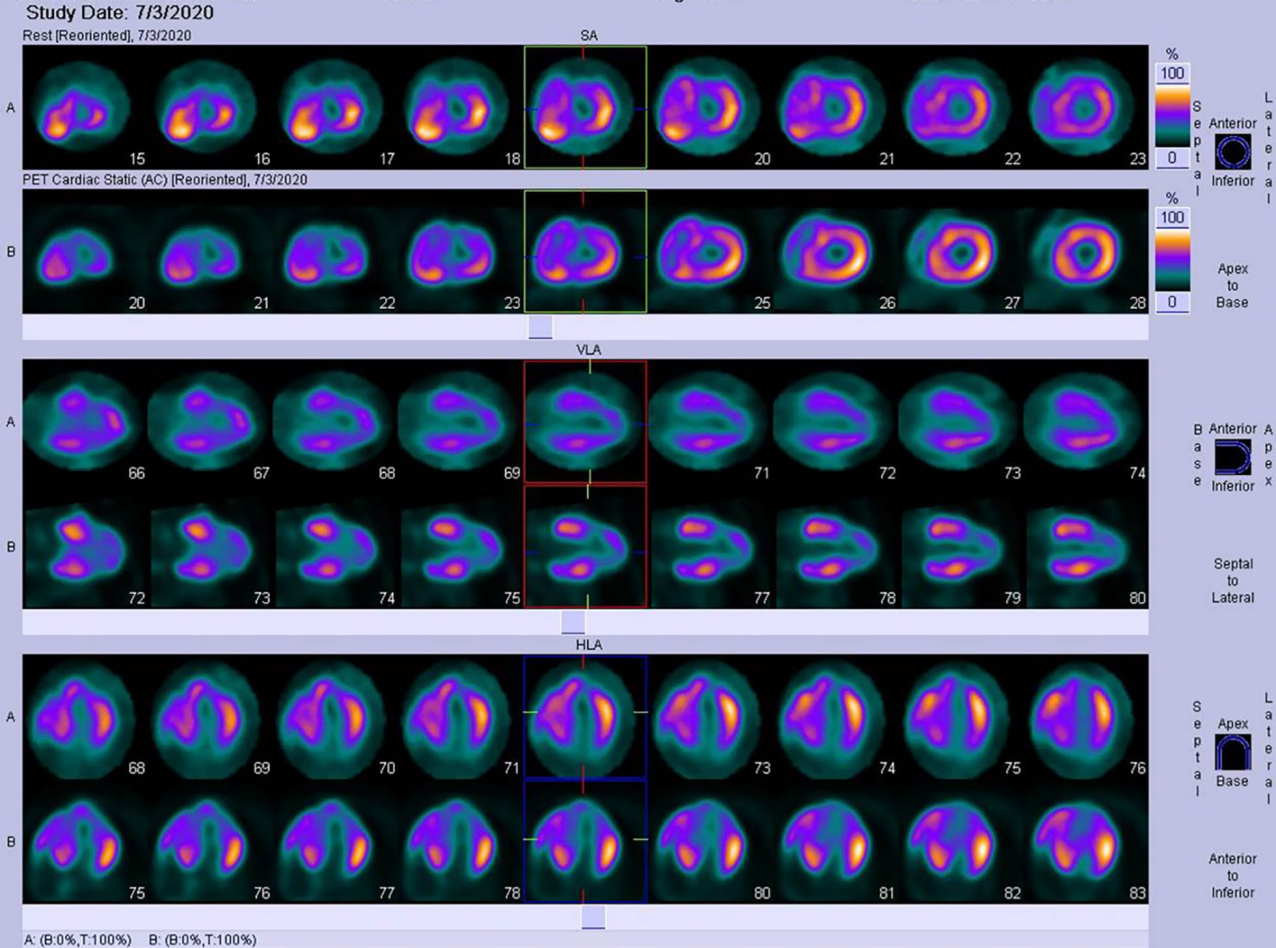
Positron emission computed tomography (PET-CT). Cardiac PET-CT showed that the myocardial infarction area was in the apex and left ventricular anterior wall

To confirm the diagnosis of AMI, we measured the change in the cardiac troponin isoform I levels measured using a nonhighly sensitive assay with an upper limit of 0.02 ng/ml. The levels increased from 0.126 to 0.443 ng/ml and 0.814 ng/ml at hours 5 and 7, respectively, peaked to 3.109 ng/ml 12 h later, and then decreased. Additionally, a follow-up ECG showed significant changes compared with the first ECG (Fig. 1)—i.e., the inverted T waves became normal in the precordial leads. The immediate echocardiogram also showed reduced anterior wall motion and wall thickening. Cardiac positron emission computed tomography showed nontransmural myocardial infarction in the left ventricular anterior wall and the apex, in accordance with the left anterior descending artery (LAD)-dominant areas (performed 9 days after admission, Fig. 3). Overall, these clues supported the diagnosis of AMI in the left ventricular anterior wall.

**Fig. 4:**
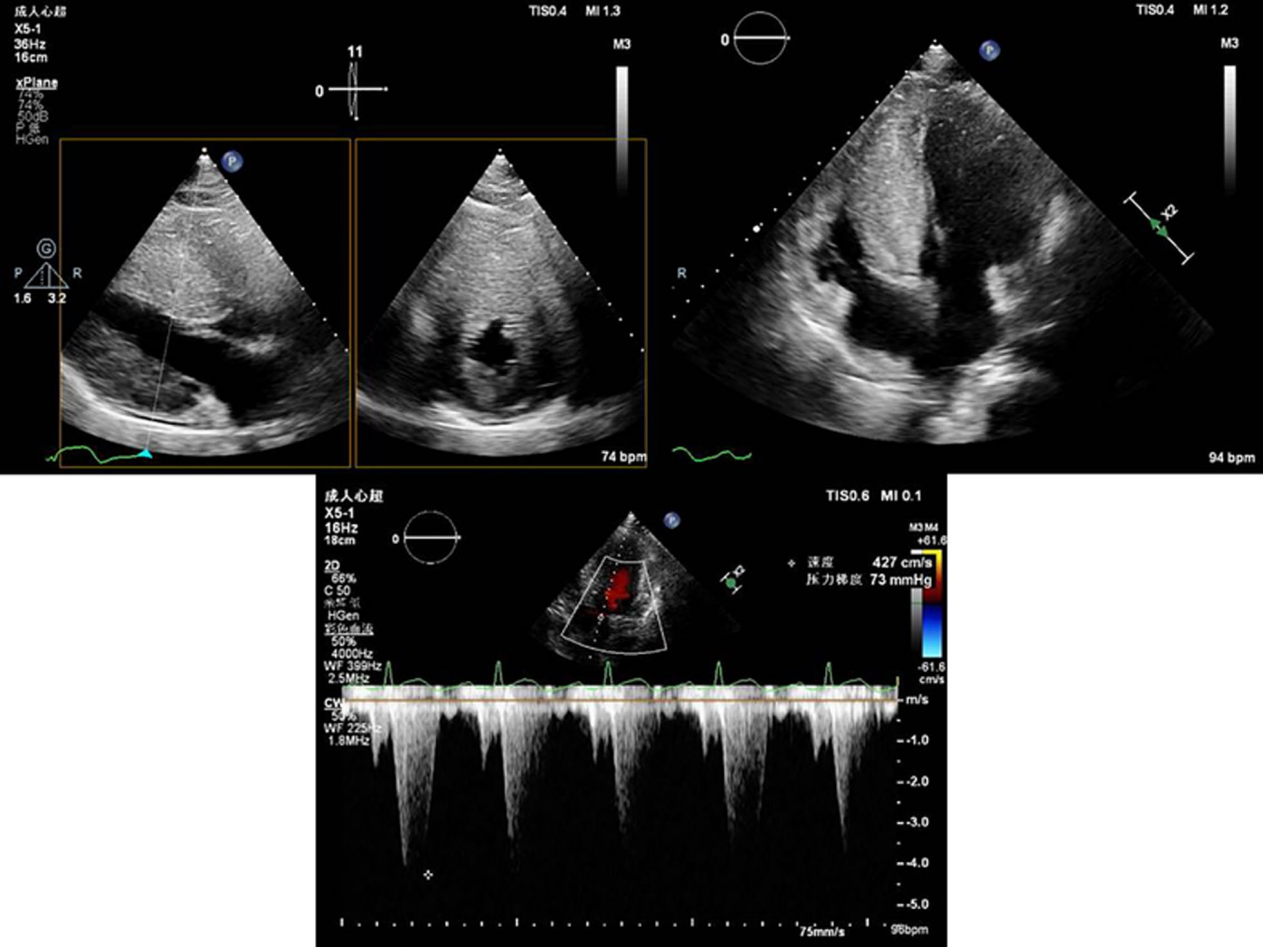
Echocardiogram. The echocardiogram demonstrated hypertrophy involving the interventricular septum, left ventricular anterior wall and apex, and severe LVOT obstruction and RVOT obstruction. The peak gradient across the LVOT was 73 mmHg, and the RVOT was 23 mmHg at rest

Further evaluation of the echocardiogram demonstrated hypertrophy involving the interventricular septum, left ventricular anterior wall and apex, severe LVOT obstruction and mild right ventricular outflow tract (RVOT) obstruction. The measured wall thickness values were as follows: 34 mm in the anterior interventricular septum, 36 mm in the apex, 9 mm in the lateral wall, 12 mm in the posterior wall, and 11 mm in the inferior wall. Systolic anterior motion (SAM) resulted in the narrowest outflow tract width of 5 mm. The peak gradient across the LVOT was 73 mmHg, and the RVOT was 23 mmHg at rest. Tissue Doppler imaging showed a mitral septal e' of 3 cm/s and mitral lateral e' of 7 cm/s. The mean E/e' was 10, and the E/A was 0.7, indicating grade 2 diastolic dysfunction. Systolic function was preserved with a left ventricular ejection fraction (LVEF) of 64% (performed 6 days later; Fig. 4).

**Fig. 5:**
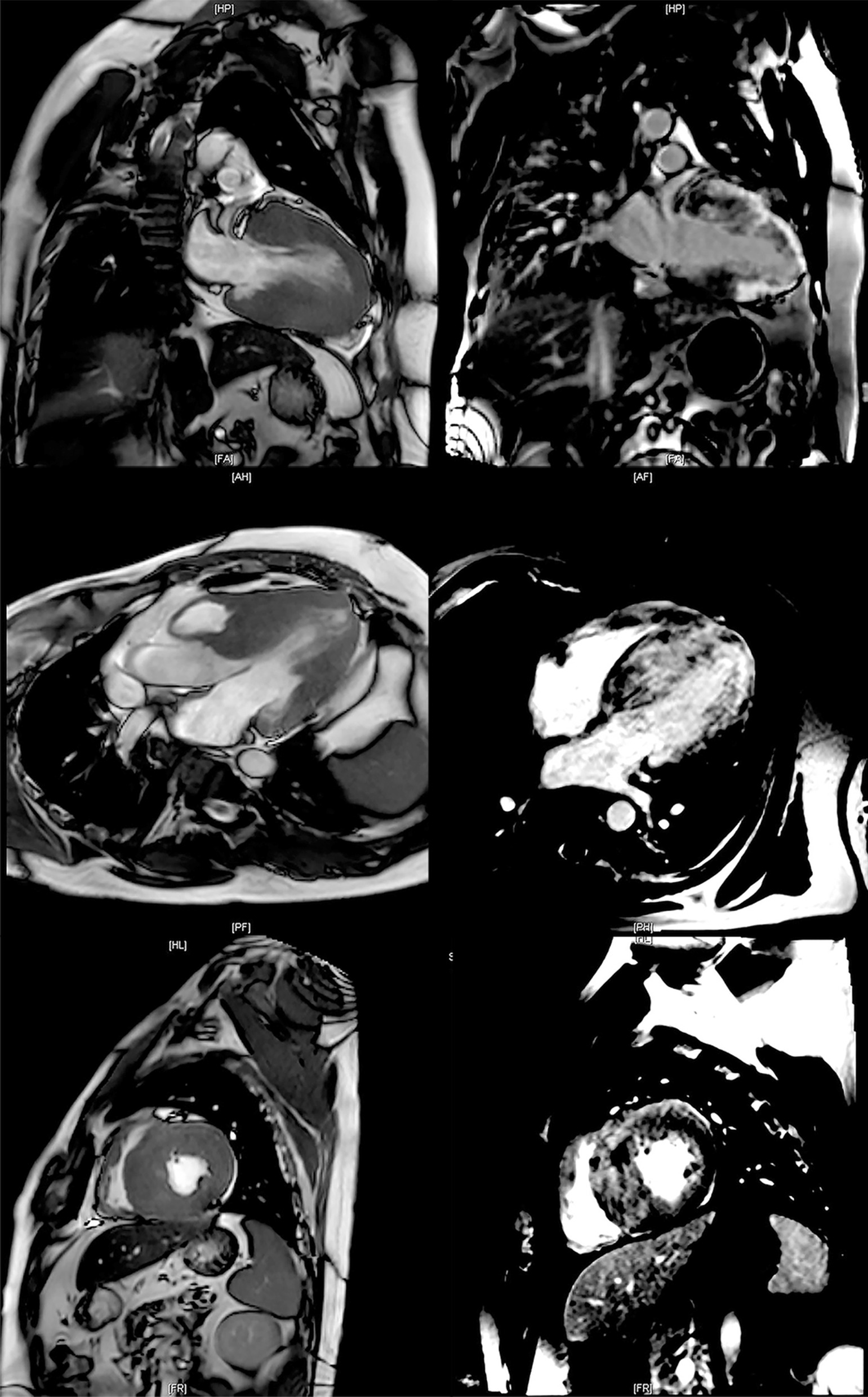
Cardiac magnetic resonance (CMR). CMR showed that the left ventricle was diffusely and extremely hypertrophied. The thickness of the interventricular septum was 40 mm. In the end diastole, the left ventricular mass was 274.71 g, and the right ventricular mass was 59.38 g. The left ventricular cavity was relatively small (diastolic diameter 39 mm) without an enlarged left atrium (anterior–posterior diameter 26 mm). The measured LVEF was 56%, and the cardiac output was 6.6 L/min. CMR showed diffusely patchy late gadolinium enhancement (LGE) in the septum, and middle and distal segments of the left ventricle. The percentage of left ventricular fibrosis was 51.6%

Cardiac magnetic resonance (CMR) imaging showed a concentric morphological subtype of HCM [14]—i.e., the left ventricle was diffusely and extremely hypertrophied, with an interventricular septum thickness of 40 mm, inferior wall thickness of 15–16 mm, anterior wall thickness of 18–19 mm, and lateral wall thickness of 12–13 mm. The trabeculae were thickened, and the anterior and posterior papillary muscles were also hypertrophied. In the end diastole, the left ventricular mass was 274.71 g, and the right ventricular mass was 59.38 g. The left ventricular (LV) cavity was relatively small (diastolic diameter 39 mm) without an enlarged left atrium (anterior–posterior diameter 26 mm). The left and right outflow tracts were narrowed with SAM and mild mitral regurgitation. The measured LVEF was 56%, and the cardiac output was 6.6 L/min. CMR showed diffusely patchy late gadolinium enhancement (LGE) in the septum, and middle and distal segments of the left ventricle. The percentage of left ventricular fibrosis was 51.6% (performed 4 days after admission; Fig. 5).

**Fig. 6:**
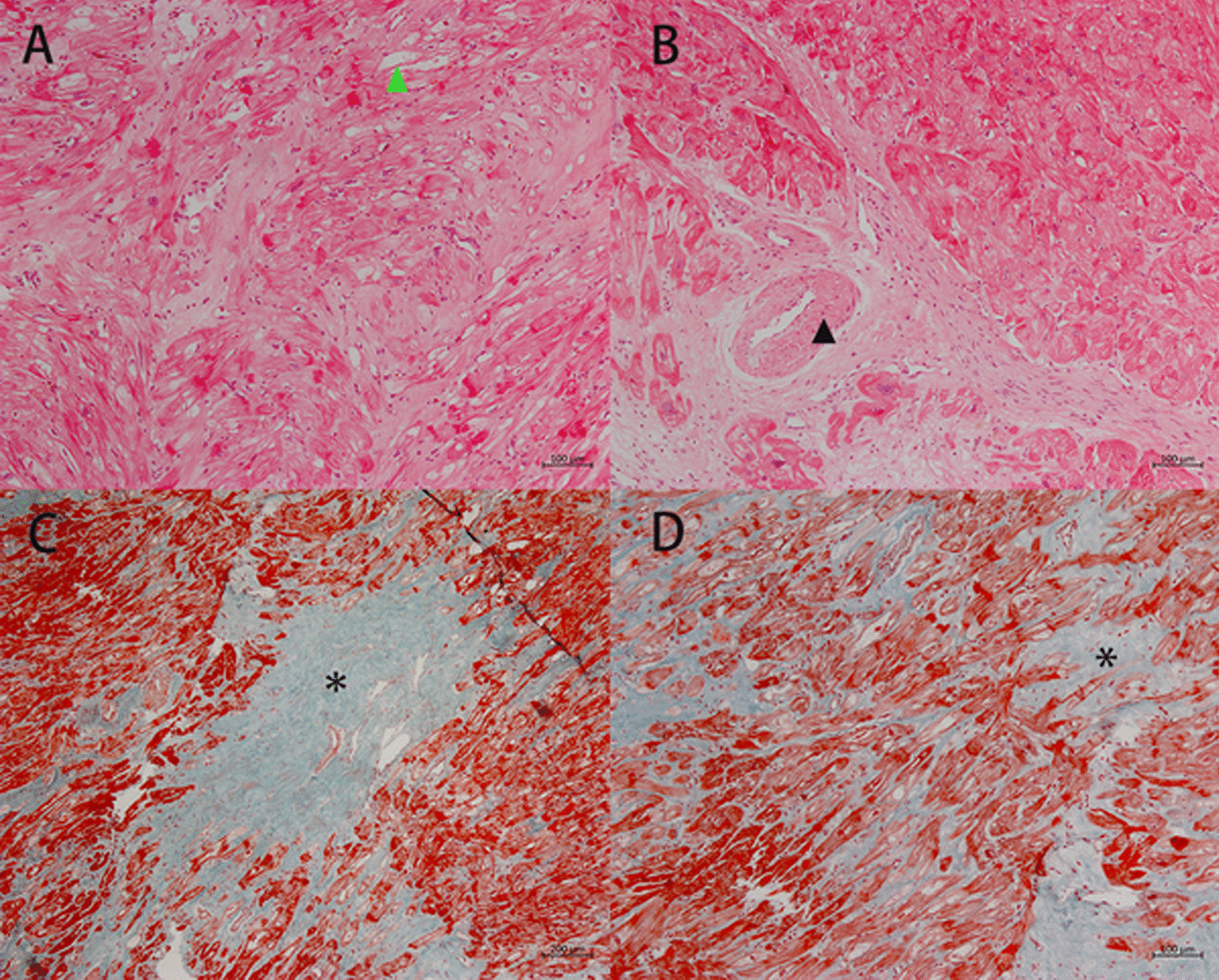
Endomyocardial biopsy. The endomyocardial biopsy revealed cardiomyocyte hypertrophy with vacuolar degeneration (green triangle in 6A), and aberrantly oriented myocytes (**a**, **d**). Interstitial fibrosis (asterisk in **d**), abnormal small vessels with thickened walls (black triangle in **b**), and focal fibrous scar formation (asterisk in **c**) were also observed. (**a**) and (**b**) are HE stained with tenfold magnification. (**c**) and (**d**) show Masson's trichrome staining at fivefold and tenfold magnifications, respectively. Biopsy images were acquired using a ZEISS Axioskop 40 Microscope and Zeiss ZEN acquisition software at a 2752 × 2208 resolution. Final image adjustments were done with Adobe Photoshop 22.0 software to produce a final size of 1200*963 pixels

The endomyocardial biopsy revealed cardiomyocyte hypertrophy with vacuolar degeneration, and aberrantly oriented myocytes. Interstitial fibrosis, abnormal small vessels with thickened walls, and focal fibrous scar formation were also observed. The endomyocardial biopsy excluded other diseases, such as phenocopy of HCM and amyloidosis (performed 2 weeks after admission; Fig. 6).

**Fig. 7:**
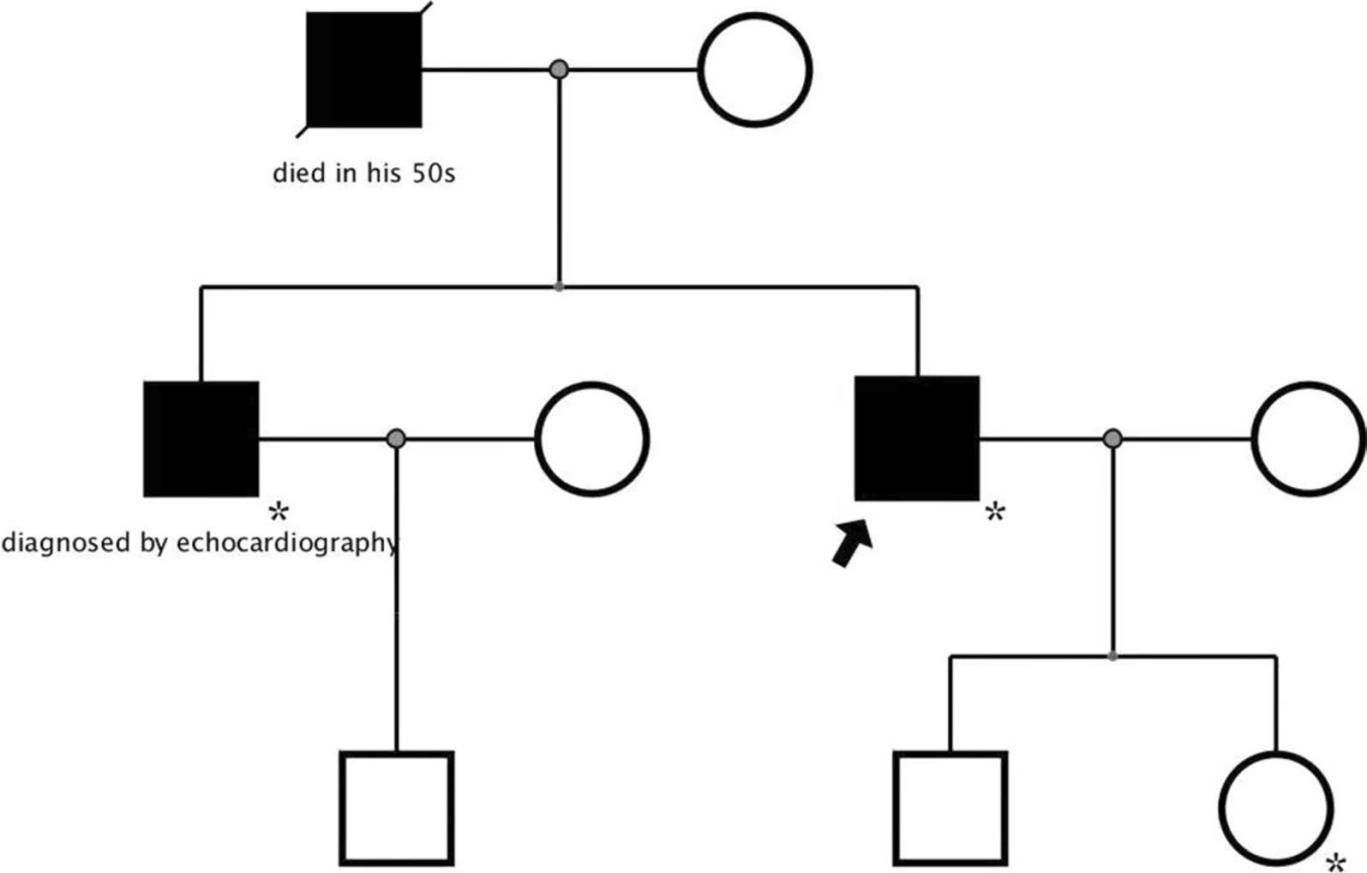
The pedigree. The patient and his older brother had HCM phenotype and survived. His father died of HCM in his 50 s. Other family members were healthy. The gene KCNJ2 p.V93I heterozygous variant was carried by the patient, his 10-year-old daughter and his older brother (labeled with asterisks)

Genetic testing revealed that the patient carried a KCNJ2 p.V93I heterozygous variant, which was classified as a variant of uncertain significance based on the American College of Medical Genetics and Genomics (ACMG) guideline. No sarcomere gene mutations or variants were found. The gene variant was also carried by his 10-year-old daughter and older brother. His daughter was healthy with a normal ECG and echocardiogram (the pedigree; Fig. 7).

Finally, we gave the patient a diagnosis of obstructive HCM, with extreme hypertrophy, biventricular obstruction and diffuse myocardial fibrosis complicated with MINOCA, which is a rare phenotype. His treatment was also a dilemma. On the one hand, he was at high risk of sudden cardiac death because of severe hypertrophy of the left ventricle (> 30 mm) and biventricular obstruction. On the other hand, diffuse fibrosis and new AMI in the anterior wall increased the risk of septal myectomy, such as post-operative cardiac dysfunction, complete heart block and in-hospital mortality. After prudent discussion, extended septal myectomy (Morrow Procedure) was performed one month later. The patient recovered well during the follow-up three months after the surgery.

## Discussion

We report the case of a patient with HOCM presenting with MINOCA. Despite the presence of AMI and diffuse fibrosis, extended septal myectomy was performed to relieve ventricular outflow tract obstruction and the patient’s symptoms. This case highlighted that treatment for MINOCA in HOCM should be individualized based on the patients’ specific conditions, including the infarct size, obstructive severity, and fibrosis level.

The diagnosis of MINOCA requires an ischemic basis for the clinical presentation—i.e., a diagnosis of AMI should be made first [3]. Specifically, the universal AMI definition requires clinical evidence of myocardial necrosis and ischemia [15]. In this case, typical symptoms, ischemic ECG changes, and regional wall motion abnormalities were evidence of acute myocardial ischemia, while dynamic troponin I release and infarcted nuclear imaging supported myocardial necrosis. One distinct characteristic is that ST-segment elevation was always present in precordial V1-V6 leads in this patient. During an acute angina attack, this phenomenon could be misleading and confusing. However, a few days later, T-wave inversion, a sign of postischemic stunning, was observed in these precordial leads (LAD distributed). One shortcoming of this case report was that we did not perform optical coherence tomography (OCT) or intravascular ultrasound (IVUS) imaging to confirm the evidence of nonobstructive atherosclerosis, plaque rupture and erosion on the angiogram [3]. However, the interventional cardiologist confirmed that obstructive disease was not overlooked, as shown in the coronary angiogram.

Previously, some case reports also demonstrated AMI in HCM with a normal coronary artery or diminutive coronary artery [5–7]. However, MINOCA in HCM is rare and requires further study to uncover the underlying mechanism [7]. The most likely reason is that ischemia results from an imbalanced oxygen supply and demand at the microvascular level without acute epicardial obstruction [8]. The pathophysiological changes include distortion of the arteriolar architecture, intramural small vessel disease, inadequate capillary density in relation to the increased myocardial mass, and impairment of endothelium-dependent vasodilation [8–10]. Additionally, LVOT obstruction might enhance thrombin generation and platelet activity, accounting for micro or transient thrombus/embolism formation in the coronary artery [13]. Another suggested mechanism is that in HOCM, increased wall stress leads to coronary spasm and squeezing, and decreased coronary flow, causing microvascular disorder and myocardial infarction [12]. In this patient, extreme hypertrophy combined with biventricular obstruction may have resulted in a compromised blood supply of the LAD branch that manifested as slow blood flow.

In HCM patients, the prevalence of significant epicardial coronary artery disease (CAD) was up to 20% [9]. However, the incidence and prevalence of AMI in the HCM population, which could be caused either by obstructive epicardial CAD or MINOCA, have not been investigated in large studies. Theoretically, HCM with MINOCA is different from HCM with AMI caused by obstructive CAD. Therefore, their prognoses are considered different. However, no studies have compared the two phenotypes. Nevertheless, a previous study showed that adult HCM patients with concomitant severe CAD are at a higher risk of death than their counterparts [9]. In summary, coronary angiography is necessary for HCM patients with concomitant AMI to clarify the underlying cause and determine the following treatment plan.

Patients with HOCM and MINOCA are challenging to treat, particularly in the presence of a large area of myocardial infarction. First, the infarcted myocardium cannot be saved by revascularization, because no epicardial coronary stenotic lesions exist. Additionally, no effective treatments are currently available to reverse myocardial infarction mediated by other mechanisms. Second, the healthy viable myocardium is significantly reduced once massive myocardial infarction occurs. As shown in this case, the presence of biventricular obstruction makes the clinical scenario more complex. On the one hand, if septal reduction therapy is performed, part of the healthy viable myocardium may be cut off, and the normal viable myocardium will be reduced further, possibly leading to cardiac dysfunction after surgery. On the other hand, if septal reduction therapy is not performed, patients will continue to show severe LVOT obstruction and have a high risk of sudden death. Biventricular obstruction will also aggravate myocardial ischemia and worsen cardiac function. No consensus exists regarding the choice of treatment for these patients [16]. Individualized treatment is the focus for HCM patients. More studies on treatment is needed for this population in the future. In this case, the final decision was to perform extended septal myectomy to solve the current main problem of ventricular outflow tract obstruction. This not only relieved outflow tract obstruction and symptoms but also reduced the rate of sudden death and avoided left ventricular dysfunction after surgery [17–19].

In addition to MINOCA, this patient showed diffuse myocardial fibrosis as indicated by LGE on CMR. According to previous studies, LGE is identified in approximately 60% of the adult overt HCM population [20]. The molecular pathways leading to myocardial fibrosis remain incompletely understood. LV hypertrophy may be an important factor associated with LGE because LGE is seldom observed in mutation carriers without LV hypertrophy [21–23]. Recent studies have demonstrated that the maximal LV wall thickness is the only independent predictor of the presence and extent of LGE [23]. Myocardial fibrosis displayed on LGE might be the manifestation of ischemia. Whether patients with severe hypertrophy have recurrent ischemic attacks and/or focal infarctions is unknown. However, LGE has been extensively studied and found to be associated with adverse outcomes [20, 24]. Thus, this extremely hypertrophic and biventricular obstructive HCM patient, with extensive myocardial fibrosis superimposed by MINOCA, might have the worst prognosis.

This patient had a family history of HCM, and the genetic test results suggested a heterozygous variant of uncertain significance in KCNJ2, which encodes the Kir2.1 potassium channel [25]. This gene has not been previously reported to be associated with HCM. Previously, this gene variant was reported to enhance the function of Kir2.1 and was related to atrial fibrillation [26]. The relationship between this gene variant and HCM phenotype remains unclear. Further basic studies are required to verify the variant’s function.

In summary, this patient with extremely hypertrophic and biventricular obstructive HCM had a MINOCA attack superimposed on extensive myocardial fibrosis. We made individualized treatment decisions according to the characteristics of the patient. Septal reduction surgery was performed cautiously in this scenario to improve the patient’s symptoms.

## Data Availability

All the data presented in this case are included in this published article. Coronary angiograms are available from the corresponding author on reasonable request.

## Abbreviations

AMI: Acute myocardial infarction
CAD: Coronary artery disease
CMR: Cardiac magnetic resonance
ECG: Electrocardiogram
HCM: Hypertrophic cardiomyopathy
HOCM: Hypertrophic obstructive cardiomyopathy
LAD: Left anterior descending artery
LGE: Late gadolinium enhancement
LV: Left ventricular
LVEF: Left ventricular ejection fraction
LVOT: Left ventricular outflow tract
MINOCA: Myocardial infarction in the absence of obstructive coronary artery disease
PET-CT: Positron emission computed tomography
RVOT: Right ventricular outflow tract
SAM: Systolic anterior motion

## References

[1] Smilowitz N, Mahajan A, Roe M (2017). Mortality of myocardial infarction by sex, age, and obstructive coronary artery disease status in the ACTION Registry-GWTG (Acute Coronary Treatment and Intervention Outcomes Network Registry-Get With the Guidelines). Circul Cardiovasc Qual Outcomes.

[2] Pasupathy S, Air T, Dreyer R, Tavella R, Beltrame J (2015). Systematic review of patients presenting with suspected myocardial infarction and nonobstructive coronary arteries. Circulation.

[3] Tamis-Holland J, Jneid H, Reynolds H (2019). Contemporary diagnosis and management of patients with myocardial infarction in the absence of obstructive coronary artery disease: a scientific statement from the American Heart Association. Circulation.

[4] Agewall S, Beltrame J, Reynolds H (2017). ESC working group position paper on myocardial infarction with non-obstructive coronary arteries. Eur Heart J.

[5] Patanè S, Marte F, Di Bella G, Chiribiri A (2009). Acute myocardial infarction with diminutive right coronary artery and obstructive hypertrophic cardiomyopathy without significant coronary stenoses. Int J Cardiol.

[6] Limongelli G, Calabro' P, Pacileo G, Santoro G, Calabro' R (2007). Myocardial infarction in a young athlete with non-obstructive hypertrophic cardiomyopathy and normal coronary arteries. Int J Cardiol.

[7] Moscatelli S, Nardi B, Indolfi E (2019). P586An unusual phenocopy of hypertrophic cardiomyopathy: a case report. Eur Heart J Cardiovasc. Imaging.

[8] Marian AJ, Braunwald E (2017). Hypertrophic cardiomyopathy: genetics, pathogenesis, clinical manifestations, diagnosis, and therapy. Circ Res.

[9] Sorajja P, Ommen SR, Nishimura RA, Gersh BJ, Berger PB, Tajik AJ (2003). Adverse prognosis of patients with hypertrophic cardiomyopathy who have epicardial coronary artery disease. Circulation.

[10] Schwartzkopff B, Mundhenke M, Strauer BE (1998). Alterations of the architecture of subendocardial arterioles in patients with hypertrophic cardiomyopathy and impaired coronary vasodilator reserve: a possible cause for myocardial ischemia. J Am Coll Cardiol.

[11] Yetman AT, McCrindle BW, MacDonald C, Freedom RM, Gow R (1998). Myocardial bridging in children with hypertrophic cardiomyopathy—a risk factor for sudden death. N Engl J Med.

[12] Ushikoshi H, Okada H, Morishita K (2015). An autopsy report of acute myocardial infarction with hypertrophic obstructive cardiomyopathy-like heart. Cardiovasc Pathol.

[13] Dimitrow P, Undas A, Bober M, Tracz W, Dubiel J (2008). Obstructive hypertrophic cardiomyopathy is associated with enhanced thrombin generation and platelet activation. Heart (British Cardiac Society).

[14] Neubauer S, Kolm P, Ho CY (2019). Distinct subgroups in hypertrophic cardiomyopathy in the NHLBI HCM Registry. J Am Coll Cardiol.

[15] Thygesen K, Alpert JS, Jaffe AS (2018). Fourth universal definition of myocardial infarction (2018). Circulation.

[16] Pelliccia F, Alfieri O, Calabrò P (2020). Multidisciplinary evaluation and management of obstructive hypertrophic cardiomyopathy in 2020: towards the HCM Heart Team. Int J Cardiol.

[17] Ommen SR, Maron BJ, Olivotto I (2005). Long-term effects of surgical septal myectomy on survival in patients with obstructive hypertrophic cardiomyopathy. J Am Coll Cardiol.

[18] Liebregts M, Vriesendorp PA, Mahmoodi BK, Schinkel AF, Michels M, ten Berg JM (2015). A systematic review and meta-analysis of long-term outcomes after septal reduction therapy in patients with hypertrophic cardiomyopathy. JACC Heart Fail.

[19] Rigopoulos AG, Ali M, Abate E (2019). Review on sudden death risk reduction after septal reduction therapies in hypertrophic obstructive cardiomyopathy. Heart Fail Rev.

[20] Green JJ, Berger JS, Kramer CM, Salerno M (2012). Prognostic value of late gadolinium enhancement in clinical outcomes for hypertrophic cardiomyopathy. JACC Cardiovasc Imaging.

[21] Ho CY, López B, Coelho-Filho OR (2010). Myocardial fibrosis as an early manifestation of hypertrophic cardiomyopathy. N Engl J Med.

[22] Rowin EJ, Maron MS, Lesser JR, Maron BJ (2012). CMR with late gadolinium enhancement in genotype positive-phenotype negative hypertrophic cardiomyopathy. JACC Cardiovasc Imaging.

[23] Axelsson Raja A, Farhad H, Valente AM (2018). Prevalence and progression of late gadolinium enhancement in children and adolescents with hypertrophic cardiomyopathy. Circulation.

[24] Weng Z, Yao J, Chan RH (2016). Prognostic value of LGE-CMR in HCM: a meta-analysis. JACC Cardiovasc Imaging.

[25] Zaritsky JJ, Redell JB, Tempel BL, Schwarz TL (2001) The consequences of disrupting cardiac inwardly rectifying K(+) current (I(K1)) as revealed by the targeted deletion of the murine Kir2.1 and Kir2.2 genes. J Physiol 533(Pt 3): 697–710. 10.1111/j.1469-7793.2001.t01-1-00697.x PMC2278659 11410627

[26] Xia M, Jin Q, Bendahhou S, et al. A Kir2.1 gain-of-function mutation underlies familial atrial fibrillation. Biochem Biophys Res Commun 2005; 332(4): 1012–9. 10.1016/j.bbrc.2005.05.054 15922306

